# Road Traffic Noise Exposure and Depression/Anxiety: An Updated Systematic Review and Meta-Analysis

**DOI:** 10.3390/ijerph16214134

**Published:** 2019-10-27

**Authors:** Angel M. Dzhambov, Peter Lercher

**Affiliations:** 1 Department of Hygiene and Ecomedicine, Faculty of Public Health, Medical University of Plovdiv, 4002 Plovdiv, Bulgaria; 2 Institute for Highway Engineering and Transport Planning, Graz University of Technology, 8010 Graz, Austria or

**Keywords:** environmental noise, mental disorders, mental health, transportation noise

## Abstract

Unlike other World Health Organization evidence reviews, the systematic review on mental disorders could not provide a quantitative estimate of the effect of environmental noise. With that in mind, we aimed to update it with additional studies published through to 18 August 2019 in order to allow for a formal meta-analysis of the association of residential road traffic noise with anxiety and depression. The quality effects and random effects estimators were used for meta-analysis and the robustness of findings was tested in several sensitivity analyses. Ten studies were included in the qualitative synthesis, from which we extracted 15 estimates for depression (n = 1,201,168) and five for anxiety (n = 372,079). Almost all studies were cross-sectional and the risk of bias in them was generally high. We found 4% (95% CI: −3%, 11%) higher odds of depression and 12% (95% CI: −4%, 30%) of anxiety associated with a 10 dB(A) increase in day–evening–night noise level (L_den_). Both models suffered from moderate heterogeneity (55% and 54%), but there was evidence of publication bias only in the depression model. These findings were robust with no evidence of study-level moderators. A sensitivity analysis on an alternative set of categorically-reported estimates supported a linear relationship between L_den_ and depression. Taking into account an overall quality assessment for the included studies, we conclude that there is evidence of “very low” quality that increasing exposure to road traffic noise may be associated with depression and anxiety.

## 1. Introduction

Recent World Health Organization (WHO) evidence reviews, published in the International Journal of Environmental Research and Public Health, have revealed sufficient evidence that sources of transportation noise lead to serious annoyance [1], sleep disturbance [2], and cardio-metabolic disorders [3]. Less convincing evidence and no exposure-response information was provided for mental health [4]. Mental health is a perquisite for well-being and participation in social and economic life, with the burden already amounting to about 4% of the gross domestic product across European Union countries [5]. Unlike other chronic diseases, mental disorders such as anxiety and depression show an early-life onset [6,7], ranging from mild and time-limited to chronic and severe impairments. This feature makes the illness difficult to identify, and therefore, it is often poorly treated and overlooked [8,9,10]. The 12-month point prevalence of anxiety and depressive disorders in Western countries sums up to 10%–14% on average now [5,11], and it has become a public health priority issue around the World in the case of adults [12,13] and children [7]. Although the treatment of both conditions is increasing, undertreatment is still prevalent [8] and treatment effectiveness is modest [10]. Moreover, the pathway to mental disorders is a complex and interactive process, and over-reliance on individual treatment at the expense of broader “structural prevention” approaches [14] has further hindered the successful reduction of disease prevalence.

It was unfortunate that the WHO evidence review on mental health was hampered mainly by the broad outcome selection, which embraced different levels of severity, from health-related quality of life to clinical outcomes like depression or anxiety disorders [4]. The small number of studies in the sub-section outcomes and the diverse measurement instruments used in the available studies (symptom questionnaires, clinical diagnoses, psychotropic medication) prevented the authors from conducting full meta-analyses. Clark and Paunovic [4] concluded that there were too few studies of clinically significant mental health outcomes, and studies of large samples were needed. However, quantitative evidence is a necessary requirement for burden of disease estimation and setting exposure guidelines.

In the meantime (the mental health review included papers up to October 2015), a sufficient number of new studies on more severe mental health outcomes, such as depression and other mental health disorders, were published. A few studies were of longitudinal design and improved the quality aspect of the evidence base [15,16]. With that in mind, we aimed to supplement the WHO review [4] by providing a formal meta-analysis with these additional studies. By restricting to more confined clinical outcomes, such as depression and anxiety, and the exposure to residential road traffic noise (as the most prevalent type of traffic noise exposure) too many analysis subsets can be avoided. The updated meta-analysis should add to the growing collection of up-to-date systematic reviews on health effects of traffic noise (e.g., birth outcomes [17], cardiovascular and metabolic disease [3], and behavioral/emotional disorders in youth [18]).

## 2. Materials and Methods

### 2.1. Systematic Review Protocol

The literature searches were carried out independently by both authors, following the preferred reporting items for systematic reviews and meta-analyses (PRISMA) [19] guidelines. Only minor disagreements needed to be resolved by discussion.

We considered studies that we were aware of based on our expert knowledge of the respective literature on traffic noise and mental health. That list was augmented with potentially relevant studies [20,21,22,23,24,25] already included in the WHO review [4]. Further, the identification of studies published after the WHO review [4] was refined by a systematic literature search for original research published in English in the period 2015–2019 (last updated on 18 August 2019). In ScienceDirect, we limited the search to Research articles, Short communications, Correspondence, and Book chapters, with no additional filters. We also contacted the authors of potentially relevant studies [26] who could provide useful effect estimates. We searched MEDLINE (PubMed) and EMBASE (ScienceDirect) using the keyword string:

("traffic noise"[All Fields] OR "road traffic noise"[All Fields] OR "transportation noise"[All Fields] OR "environmental noise"[All Fields] OR "community noise"[All Fields] OR "noise exposure"[All Fields]) AND ("anxiety"[All Fields] OR "depression"[All Fields] OR "mental health"[All Fields] OR "psychiatric disorders"[All Fields] OR "mental disorder"[All Fields] OR "psychotropic medication"[All Fields] OR "antidepressants"[All Fields] OR "anxiolytics"[All Fields]).

Our intention was to narrow down the scope of our review to road traffic noise and well-defined mental disorders (anxiety and depression), for which a sufficient number of comparable effect estimates was likely to exist. The inclusion and exclusion criteria we adopted were as follows:

Inclusion criteria:

(1) Time period: 2015–August 2019;
(2) Language: English;
(3) Original research papers;
(4) Design: cohort, case-control, cross-sectional, ecological;
(5) Population: adults (≥18 years);
(6) Exposure: road traffic noise (alone or in combination with other sources);
(7) Outcome: anxiety/depression as discrete outcomes (diagnosis, psychotropic medication use, dichotomized self-reported symptoms scale);
(8) Effect size estimate: quantitative risk estimate (OR, RR, HR) and 95% CI or SE, or p-value.

Exclusion criteria:

(1) Experimental studies, review articles;
(2) Studies only including children and adolescents (< 18 years);
(3) Studies with only subjective noise ratings (e.g., annoyance or traffic intensity);
(4) Studies with no calculated or measured noise levels (e.g., only distance to source);
(5) Studies exclusively on noise sources other than road traffic;
(6) Studies with only general psychological symptoms scores;
(7) Studies with the outcomes (anxiety/depression) not dichotomized;
(8) Studies on health-related quality of life only;
(9) Studies with no quantitative data or reporting effect measures that could not be transformed.

### 2.2. Data Extraction

Information was extracted from each retrieved article on: (1) author and publication year; (2) number of distinct datasets analyzed; (3) study design and timeframe; (4) sample size and participant characteristics; (5) outcome definition and assessment; (6) exposure definition and assessment; (7) statistical analysis; (8) adjustments; (9) and adjusted effect size estimates. In some cases, we extracted the information needed from other published records of the same study.

To enable comparison with previous meta-analyses on health effects of road traffic noise [27], we extracted effect estimates rescaled to a 10 dB(A) increment in noise exposure. Most studies used the noise indicator day-evening-night noise level (L_den_); therefore, we adopted it for reporting our findings. Of note, the relationship between noise and depression/anxiety was expressed as a linear function in the main meta-analyses, therefore non-L_den_ noise metrics were not converted to L_den_ because linear regression slopes would not be affected by the absolute difference between noise metrics [28,29]. If estimates were scaled to another linear unit increase in road traffic noise [30,31,32], they were transformed as needed using the expression “exp((ln(reported effect estimate)/original unit increase)*10)”. For studies reporting categorical effect estimates across noise exposure groups [15,16,33,34] we used the “glst” or “vwls” Stata commands to linearize the risk [35], depending on whether studies reported the number of cases and the size of the total population in each exposure group e.g., [33], or only the risk estimates for each group, e.g., [15]. From some studies [15,16,30,33,34,36], alternative categorically reported risk estimates were also extracted to be used for probing a non-linear exposure-response relationship between L_den_ and depression. For the purpose of this non-linear modelling, noise levels expressed as 24-hour equivalent noise level (L_Aeq,24h_) and night noise level (L_night_) had to be converted to L_den_ using standard conversion terms (L_night_ + 8.3 dB and L_Aeq,24hr_ + 3.6 dB) derived by Brink et al. [37] for Western European countries.

Some data extraction decisions required justification. For example, Seidler et al. [36] reported two alternative models—one using the full sample and another using a reduced sample (40% of the full sample) of participants with individual socioeconomic status data available. For the main analysis, we chose the former estimate as we reckoned that the loss of representativeness would result in greater bias than the unaccounted-for confounding by socioeconomic status. Still, the reduced sample estimate was extracted for a sensitivity analysis. From Klompmaker et al. [30], we extracted the estimate unadjusted for air pollution because it referred specifically to road traffic noise and it was materially the same to the adjusted one. From He et al. [16], we used the exposure-response trend readily reported per 10 dB(A) increase in L_night_ (highly correlated with L_den_) instead of linearizing the categorically reported effect for L_den_, which would result in greater information loss. The study of Generaal et al. [32] was based on eight distinct datasets. Although the authors reported pooled effect on depression, we decided to use the estimate for each individual dataset as a separate data point in the meta-analysis; using the pooled estimate would entail bias and inadequate distribution of study weights because it would neglect the fact that we were dealing with unique populations, which systematically differed on participants’ characteristics [29]. Finally, although some studies [16,32] investigated combined noise exposure, they were retained because road traffic was the dominant noise source in the study area.

### 2.3. Risk of Bias Assessment

To ascertain the validity of eligible studies included in the qualitative synthesis, a list of predefined safeguards was used to avoid bias related to different study characteristics. This scale was adapted from the one previously developed for our updated systematic review on traffic noise and birth outcomes [38]. Higher scores indicate less bias. The criteria and scoring are given in Appendix A, Table A1. This scale was incorporated in meta-analysis algorithm and as one of the elements of the overall quality of evidence assessment.

### 2.4. Meta-Analysis

Since almost all included studies reported odds ratios (ORs), the OR along with its 95% CI was used as a common measure of effect size. We pooled linear exposure-response relationships for a 10 dB(A) increase in L_den_ with anxiety and depression. As in our previous meta-analysis [38], effect estimates were pooled under two alternative meta-analytical estimators—the random effects (RE) model and the quality effects (QE) model. The RE estimator is traditionally used in the presence of high between-study heterogeneity, but it has been scrutinized on multiple occasions for underestimating the true variance and producing overconfident results [39,40,41,42,43]. Therefore, we report our main findings under the QE model, which allowed for the inclusion of information on the risk of bias in primary studies into the estimation of meta-analysis weights [41,42]. That is, a synthetic bias variance was computed from our bias scores, ranking each study relative to the others, to adjust inverse variance weights based on the additional variance contribution from internal study biases [41]. The QE model favours larger studies with lowest probability of random error and exhibits a lesser true variance than the RE model, regardless of heterogeneity. Thereby, the QE model maintains the correct coverage probability of the confidence interval without underestimation of the statistical error [41].

We assessed the possibility of publication bias by evaluating Doi plots [44,45], a variant of the normal quantile versus effect plot using a rank-based measure of precision (Z score), instead of the standard error, and plots it against the effect size [44]. The most precise studies define the midpoint around which results scatter, whereas smaller less precise studies produce an effect size that scatters increasingly widely, and the absolute Z score gradually increases for both smaller and larger effect sizes on either side of that of the precise studies. Doi plot asymmetry was quantified with the Luis Furuya-Kanamori (LFK) index by averaging half of the sum of the Z score plus the normalized effect size across the meta-analysis [44,45]. The LFK index quantifies the difference between the two areas under the Doi plot, created by the perpendicular line to the X-axis from the effect size with the lowest absolute Z score on the Doi plot [45]. A symmetrical, mountain-like Doi plot and LFK index <|1| indicate no asymmetry, LFK index between |1| and |2|, minor asymmetry, and LFK index >|2|, major asymmetry [44].

Sensitivity analyses were pre-specified in addition to the main meta-analysis. First, we used the leave-one-out method to check the robustness of the point estimate upon exclusion of each individual estimate one-at-a-time. We then carried out subgroup meta-analysis for depression according to the study characteristics “outcome assessment method (diagnosis, antidepressants, or self-report scale)” and “noise source (road traffic noise only or multiple sources)”. We employed meta-regressions to test for between-subgroup heterogeneity. For that purpose, the dataset was exported to Stata, where we used the “regress” command with robust standard errors (Huber-Eicker-White-sandwich) and the QE weights as analytic weights [44]. We also conducted meta-regressions of the continuous study-level factors “mean/median age”, “minimum age”, “percentage of female participants”, “sample size”, “mean/median noise level”, and “prevalence of depression”.

Next, in a subset of studies [15,16,30,33,34,36], we tested the exposure-response relationship between L_den_ and depression for non-linearity by fitting a restricted cubic spline model. We used the inverse variance weighted least squares regression with cluster-robust error variances (REMR model). It is a one-step procedure that requires no knowledge of the correlation structure of the regression coefficients because it stacks included effects as a cluster by study and uses the cluster-robust analysis to obtain a robust standard error. Thus, it treats observations as independent across clusters but correlated within each cluster [46,47]. Since the reference noise exposure category varied across studies, we first converted the absolute noise level (after all metrics had been converted to L_den_) to noise increments from the reference category by subtracting the reference category from the non-reference category for each reported non-reference effect (i.e., noise increments started from zero and were later back-transformed for clear visual representation). Detailed theoretical rationale and Stata codes behind this method can be found in the methodological paper of Xu and Doi [47].

Statistical heterogeneity was indicated by a significant Cochran’s Q at the *p* < 0.1 level and quantified by the I^2^ statistic. The I^2^ cut-offs of 25%, 50%, and 75% suggested low, moderate, and high heterogeneity, respectively [48]. Meta-analyses were conducted in MetaXL v. 5.3 (EpiGear International Pty Ltd, Sunrise Beach, Queensland, Australia) and Stata v. 13 (College Station, TX: StataCorp LP.).

### 2.5. Quality of Evidence Assessment

The quality of evidence for the effect of road traffic noise on each outcome was rated according to the Grading of Recommendations Assessment, Development and Evaluation (GRADE) system [49,50] with slight adaptations. As in previous noise and health meta-analyses, cohort and case-control studies started with a “high” quality rating because a randomized controlled trial is neither a typical nor feasible design in the field; on the other hand, analytic studies are considered the gold standard (in practical terms) [3,38]. Cross-sectional studies started with a “low” quality rating. The quality of evidence was reduced by one category, when a high risk of bias (high bias across studies), inconsistency of results (heterogeneity and disparate findings across studies), indirectness of evidence, imprecision of the effect estimate (wide 95% CI including values < 0.75 or > 1.25) [3], or publication bias were observed, or the evidence was based on only one high quality study. The quality of evidence was increased by one grade if the magnitude of the effect was large (> 1.25 or < 0.75) [3], if accounting for all plausible biases would have increased the observed effect, or if there was an exposure-response gradient (significant trend) [50].

## 3. Results

### 3.1. Literature Search Results

**Figure 1:**
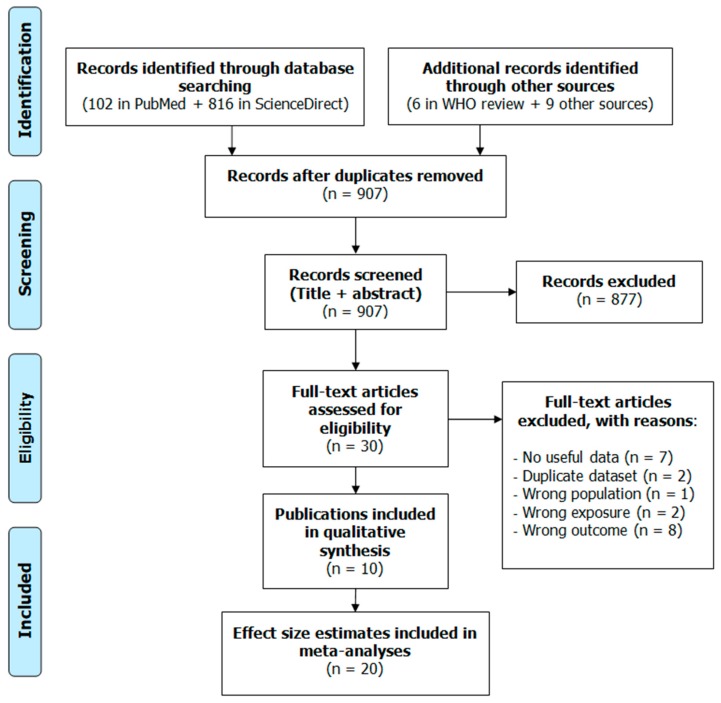
Study selection flow diagram.

The study selection flow diagram is presented in Figure 1. Database searches identified 102 records in PubMed and 816 in ScienceDirect. Searching our own records and the Internet retrieved nine publications. The WHO review [4] provided six additional publications [21,22,23,24,25,51]. After removing duplicate records, we screened the titles and abstracts of the remaining 907 records and further excluded 877 that were deemed irrelevant, leaving us with 30 full texts for in-depth review. One of them [22] did not report results for anxiolytic or antidepressant use, rather it had combined psychotropic medication use as the outcome; the outcome in the study by Bocquier et al. [21] was combined anxiolytic–hypnotic medication intake; and others had general psychological distress [23,51], quality of life [52,53], or sleep/hypnotic medication use [54,55] as the outcome. In two studies, the exposure considered was noise annoyance [56,57]. Several studies were discarded because they contained no useful quantitative data [24,58,59,60,61]. We dropped two studies because they were based on the same dataset of an already included study [62,63]. The study of Rudolph et al. [64] was excluded because it included teenage participants only. Ultimately, due to the different inclusion/exclusion criteria, only one of the WHO evidence review papers [20] was retained. Thus, 10 publications were finally included in the qualitative synthesis [15,16,20,30,31,32,33,34,36,65], with some of them supplying more than one effect estimate. Overall, 20 estimates were used for the main meta-analyses, and six publications provided additional categorical effect estimates for the non-linear meta-analysis. 

### 3.2. Narrative Description of the Studies Included

Table 1 shows abstracted descriptive characteristics of the studies included in the systematic review. Of the 10 publications, seven reported results from cross-sectional studies [20,30,31,32,33,34,65], one from a case-control study [36], and two from cohort studies [15,16]. Of note, Generaal et al. [32] analyzed eight distinct datasets; therefore, we treated them as independent studies. All studies but one [16] were conducted in Europe, mostly in the Netherlands.

Sample sizes varied from moderate to very large. Overall, response rate was < 60%. Two studies were based on pregnancy cohorts and included only female participants of relatively young age [16,32]. Five studies included only middle-aged and elderly participants [15,20,32,36], and Zock et al. [65] included Dutch from all age groups (0 to > 65 years). Generaal et al. [32] used data from several specific populations, such as the psychiatric cohort in NESDA. Thereof, studies with limited representativeness of the general adult population were penalized and received lower quality score (Table A2).

Eight publications reported results (15 estimates) for depression [15,16,20,30,32,33,36,65] and five for anxiety [20,30,31,33,65]. Four of the depression studies had data on clinically diagnosed depression [16,32,36,65], four relied on antidepressant use as a proxy for depression [15,20,30,33], and the rest used self-report symptoms scales. As for anxiety, all studies had data on either clinical diagnosis or anxiolytic medication use.

All studies calculated road traffic noise exposure from a European Union noise map or by another valid method. The majority of studies calculated noise level at the most exposed façade, but Zock et al. [65] and Leijssen et al. [34] calculated noise at the postal code-level, and He et al. [16] relied on a land-use regression model. Most studies considered road traffic noise as a separate exposure, while He et al. [16], Generaal et al. [32], and Leijssen et al. [34] considered multiple traffic noise sources combined. Noise modelling was generally of moderate accuracy—i.e., propagation modelling (engineering method) was used with input data of an acceptable quality and with a consideration of noise barriers [15,32,33,36]—but only one study considered participant’s dwelling floor [15], and none conducted validation measurements. L_den_ was the indicator of choice in most studies, with the exception of Floud et al. [20] and Seidler et al. [36] who reported L_Aeq,24h_ and He et al. [16] where the linear trend was reported for L_night_. In most studies, data on noise exposure preceding the study period were available, and Seidler et al. [36] went further by conducting a sensitivity analysis in a restricted sample of long-term (> 10 years) residents.

Statistical methods were largely compatible across studies—most authors used logistic regressions and reported ORs, as their studies were cross-sectional. Most of the studies considered important confounding factors, including age, sex and education or socioeconomic status. Orban et al. [15] also had information on area-level socioeconomic status, and Klompmaker et al. [30] and Leijssen et al. [34] considered various individual- and area-level confounders. However, some studies also adjusted for potential mediators of the association between noise and mental health [16,20,33,34], which we view as a source of bias. Additional bias was suspected for six studies, which reported effect estimates needing transformation or being associated with noise from multiple traffic sources. According to the quality scores presented in Table A2, least bias was suspected for the study of Seidler et al. [36], followed by Zock et al. [65], He et al. [16], and Generaal et al. (NEMESIS dataset) [32], whereas the study of Floud et al. [20] received the lowest quality score.

### 3.3. Meta-Analysis for Depression

**Figure 2:**
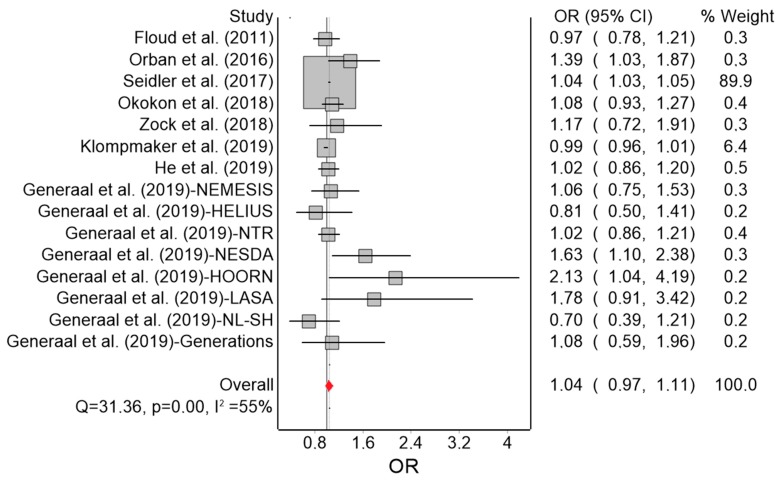
Forest plot showing the effect of a 10 dB(A) increase in road traffic noise level on depression under the quality effects model (OR: odds ratio, CI: confidence interval, Q and I^2^: heterogeneity statistics).

**Figure 3:**
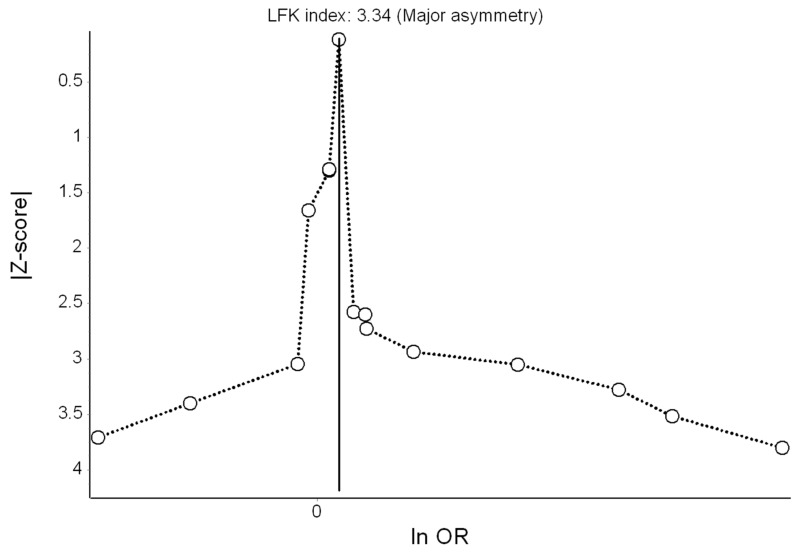
Doi plot showing the risk of publication bias in the meta-analysis of the association between road traffic noise and depression (ln OR: log odds ratio, LFK: Luis Furuya-Kanamori index).

The results of the QE meta-analysis for depression are shown in Figure 2. Based on 15 estimates, a 10 dB(A) increase in L_den_ was (marginally) associated with a 4% higher odds of depression. Heterogeneity in the model was moderate. Visual inspection of the Doi plot indicated major asymmetry (Figure 3) and the high LFK index (3.34) suggested publication bias was likely. Under the RE model, the effect remained virtually the same (OR = 1.04; 95% CI: 0.99, 1.09). In the leave-one-out meta-analysis, the pooled effect estimates also remained robust. Using the alternative effect estimate from Seidler et al. [36] (from the model with the reduced sample with socioeconomic status data), no relevant change was observed (OR = 1.05; 95% CI: 0.97, 1.13; I^2^ = 61%).

**Figure 4:**
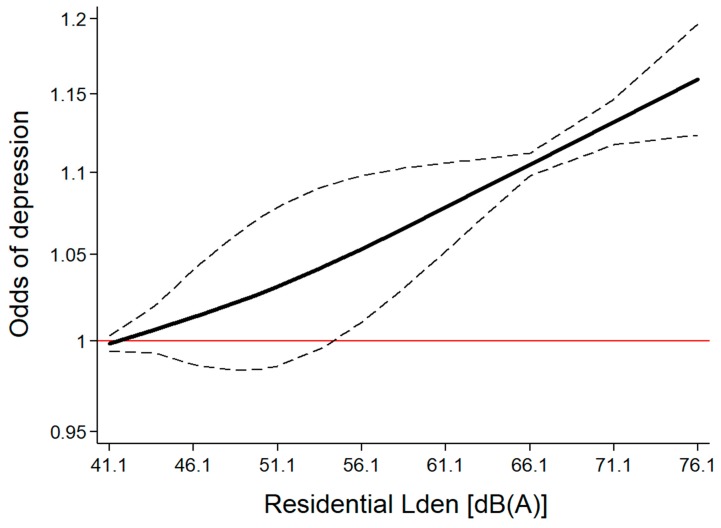
Exposure-specific relationship between day-evening-night noise level (L_den_) and the odds of depression (based on categorically reported effects in six studies).

As a next step, we generated a dataset containing categorically reported risk estimates with corresponding noise levels extracted from six studies (i.e., clusters), where noise level ranged from 41.1 to 76.1 dB(A) L_den_ (Table A3). A restricted cubic spline model was created, which generated two splines, which were then employed for the potential non-linear exposure-response modelling (Figure 4). With the REMR model (root mean squared error = 0.040), the estimated regression parameters b1 = 1.003 and b2 = 1.001 were not found to differ (test for equality of slopes: *p* = 0.823), suggesting that the linear model adequately represented the exposure-response relationship between traffic noise and the odds of depression. The effect turned statistically significant at around 55 dB(A). If more than three knots were used to build the splines, there was still no deviation from linearity (data not shown).

### 3.4. Meta-Analysis for Anxiety

**Figure 5:**
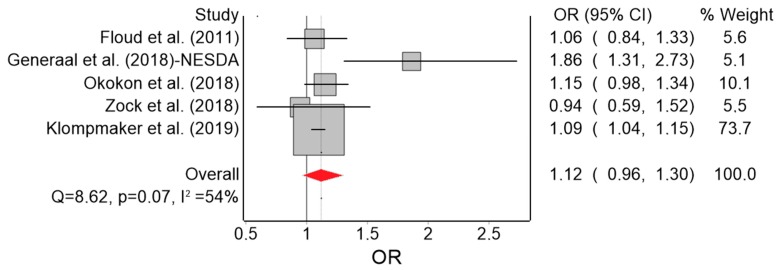
Forest plot showing the effect of a 10 dB(A) increase in road traffic noise level on anxiety under the quality effects model (OR: odds ratio, CI: confidence interval, Q and I^2^: heterogeneity statistics).

**Figure 6:**
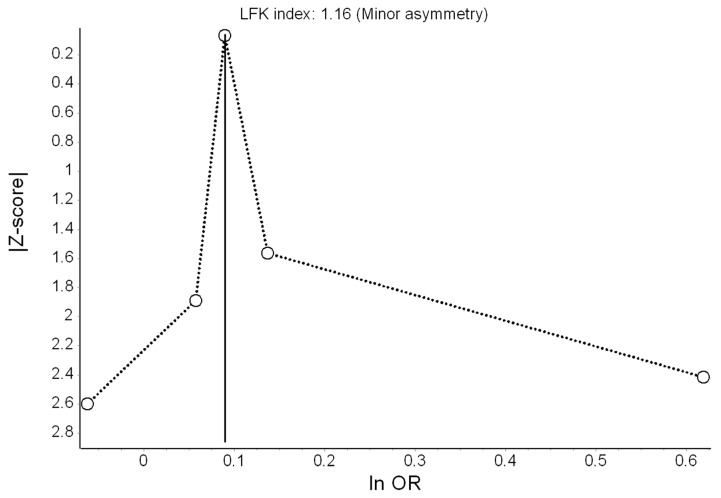
Doi plot showing the risk of publication bias in the meta-analysis of the association between road traffic noise and anxiety (ln OR: log odds ratio, LFK: Luis Furuya-Kanamori index).

Based on five effect estimates, the QE model yielded 12% higher odds of anxiety (marginally) associated with a 10 dB(A) increase in L_den_ (Figure 5). Heterogeneity in this model was moderate (I^2^ = 54%) and we found no evidence of serious publication bias (Figure 6). Under the RE model, the effect reached OR = 1.15 (95% CI: 1.01, 1.30). The only remarkable results of the leave-one-out meta-analysis were the lack of heterogeneity (I^2^ = 0%) when Generaal et al. [31] was excluded, and the higher pooled effect (OR = 1.18; 95% CI: 0.94, 1.48) when Klompmaker et al. [30] was excluded.

### 3.5. Moderators of the Effect of Road Traffic Noise on Depression

Next, we carried out subgroup meta-analysis and meta-regressions (Table 2). We found no evidence that any of the available study-level factors acted as a moderator at the *p* < 0.05 level.

### 3.6. Quality of Evidence according to GRADE 

The quality of evidence for depression was graded as “very low”; that is, the estimated effect of noise on depression was very uncertain. With most studies being cross-sectional, we started already at a “low” quality rating. This rating deteriorated further due to evidence of publication bias, high risk of bias, and inconsistent effects across studies. For anxiety, the quality of evidence was also graded as “very low”. Because of the cross-sectional design of the studies, we started with a “low” quality rating. The effect estimates were generally consistent across studies, went in the expected direction, and heterogeneity was moderate. In addition, heterogeneity was only due to one particular study, which had a modest contribution to the overall effect, and there was no evidence of serious publication bias. However, the risk of bias was high, the effect size was small (<1.25), and the upper bound of the 95% CI exceeded 1.25. 

## 4. Discussion

### 4.1. Major Findings

We systematically reviewed the literature on residential road traffic noise and depression/anxiety. Ten studies were included in the qualitative synthesis, from which we extracted 15 estimates for depression (*n* = 1,201,168) and five for anxiety (*n* = 372,079). We found 4% higher odds of depression and 12% of anxiety associated with a 10 dB(A) increase in L_den_. These effects were marginally significant. In addition, the observed relationship between L_den_ and depression appeared linear, reaching statistical significance beyond 55 dB(A). While both models suffered from moderate heterogeneity, in the anxiety model it was completely due to the Generaal et al. [31] study. Subgroup meta-analysis and meta-regressions did not reveal effect modification by the available study-level factors tested.

Overall, we found evidence of “very low” quality that increasing exposure to road traffic noise may be associated with depression and anxiety. Likewise, the WHO review [4], in which a meta-analysis and meta-regression was not employed, inferred “very low” quality evidence for an effect of road traffic noise on anxiety/depression medication intake, self-reported depression/anxiety, and interview measures of depression/anxiety. Of note, the poor quality of evidence does not mean that noise is not a risk factor for mental disorders [4]. In fact, such a relationship is both biologically and psychologically plausible [61,66,67], with mechanistic hypotheses covering stress-related pathways, increase in oxidative stress level [61], and constrained restorative and social experiences in the residential environment [67,68]. This is also in line with the findings of a recent meta-analysis on transportation noise and behavioral/emotional disorders in children and adolescents [18]. Some of the non-significant and heterogeneous findings could be attributed to studies adjusting for mediators/moderators [16,33,34] and/or poor exposure assessment [16,34]. Controlling for too many potential confounders or mistakenly adjusting for mediators [69,70,71] may underestimate the effect and should not be taken lightly.

The WHO review [4] did not include the new studies we considered, and the judgement was made without a formal meta-analysis. Another potential caveat of the WHO review was that the key confounding factors, that one would expect a study to adjust for, were not a priori reported and the quality of their assessment was not considered [4]. Moreover, the various qualities of the noise exposure assessment methods do not appear to have been thoroughly considered. Although we cannot provide a definitive solution here, we proposed an alternative scoring protocol for the bias arising from the factors believed to confound the association in question. We judged age, sex, and education/socioeconomic status as the most important confounders. Yet, these factors could be also effect modifiers, and therefore their role in causal models should be carefully examined [69,70,71]. We also refined the scoring system for the quality of noise exposure assessment because exposure misclassification in the included studies also merits consideration. In most studies, standard engineering models for noise were used, but description of traffic data used for calculation and its completeness was only partially available, and most studies did not conduct validation measurements or consider dwelling floor. Therefore, the amount and role of exposure misclassification for the obtained estimates remain unknown. Moreover, for mental health outcomes, which are often associated with severe sleep problems, the sound exposure measure of choice would probably be the L_night_ at the bedroom façade or inside the bedroom.

Another limitation of the included studies is that mental health was investigated as a direct effect of noise exposure, rather than within a contextual model in which the effects of noise are realized [72]. The pathway from traffic noise to mental disorders is a complex and interactive process involving genetic, social, and environmental factors, but sufficient information on potential mediators/moderators was unavailable in the large administrative datasets used for secondary research. It is conceivable that considering those factors in future studies may shed more light on the matter. For example, noise sensitive persons show higher annoyance responses [73,74], exhibit higher trait anxiety scores, psychiatric symptoms and reactivity to sensory stimuli in general [75,76]. Noise sensitivity is also associated with higher rates of sleep disturbance when exposed to night-time noise [77,78], which in turn is associated with a higher risk of developing depressive disorders [79,80]. These multiple mutual associations between noise, noise sensitivity, sleep disturbance, and mental disorders pose a major challenge to detect direct causal links between noise and mental health disorders. A causal interpretation is further complicated by the occurrence of other significant noise-related co-morbidities beyond insomnia, such as cardiovascular disease [81,82], cognitive decline [83] and dementia [84,85,86], although the etiological relationships (bidirectionality) remain to be uncovered [80,87].

Further, the variation through major differences in contextual factors [88], which are known moderators or potential mediators like the built environment [89,90], green space [91,92,93], and social capital [94,95] could not be evaluated. 

A few studies have used advanced statistical techniques, such as structural equation modelling, to shed more light on such potential indirect pathways e.g., [67,68]. However, none of the studies included in the current systematic review made attempts to disentangle the direct from indirect effects of traffic noise.

Finally, all studies reviewed relied on static traffic noise exposure assessment, a common drawback in environmental health research, where it is implicitly assumed that people are immobile, disregarding that they are exposed not only to their living environment, but to a multitude of environmental influences along their daily movements and through their residential relocations [96]. As a reaction to this, a novel conceptualization of mental health–environment relations has been heralded, advocating the investigation of these relationships in a life-course and exposome perspective [96,97,98].

### 4.2. Strengths and Limitations

Our systematic review has several strengths. It included additional influential studies published after the WHO review was completed, with only one of the papers originally considered in the WHO review [20] included herein. The number of estimates per outcome (five for anxiety and 15 for depression) in our review exceeded the number of studies included in the majority of meta-analyses of similar outcomes listed in the Cochrane Database of Systematic Reviews [99]. We could also conduct meta-regressions, subgroup meta-analysis, and non-linear meta-analysis for depression, which was not previously possible [18].

Incorporating information on study quality in meta-analysis weights has been recommended over quality stratification, which can induce a spurious association between effect size and precision within stratum (collider-stratification bias) [100]. As in our updated systematic review on noise and birth outcomes [38], we reported results under two estimators—the RE model, which readers and experts have grown to expect, and the QE model, which outperforms the RE model in the presence of high between-study heterogeneity. Moreover, quality effects modelling has fewer limitations than other adjustment methods in meta-analysis [101]. Though, since heterogeneity in our models was only moderate, the differences between the QE and RE estimators were materially small.

This work is not without limitations. First, although we pooled a decent number of estimates for depression, with five estimates for anxiety we could not conduct meta-regressions and subgroup meta-analyses. Second, the studies we pooled together differed in terms of outcome definition and did not always use clinical diagnoses to define the outcome. Some utilized information on psychotropic medication intake, which is fairly common in environmental noise epidemiology [102], while others relied on self-report symptoms scales. Some of the studies also reported results for combined traffic noise exposure, although road traffic was the dominant noise source in the study area. To account for these methodological discrepancies, we penalized studies that did not consider clinical diagnoses of depression/anxiety and/or road traffic noise alone. We found no differences in the pooled effect on depression across subgroups defined by the mode of exposure and outcome assessment.

Third, some of the datasets included covered specific population groups such as pregnant women and older adults e.g., [16,32]. Their limited representativeness of the general adult population was addressed by penalizing them with lower quality scores and by excluding those studies from the meta-analysis to see how that would affect the results. The re-calculated pooled effect did not change the overall picture. 

Fourth, the risk estimates reported in the two cohort studies [15,16] were pooled together with odds ratios reported in the other studies, owing to the insufficient information reported for a reliable transformation. Given that those studies [15,16] contributed modestly to the pooled effect, we do not believe that has influenced our findings. 

Finally, our literature searches were limited to papers published in English and we specifically focused on road traffic noise and depression/anxiety. This leaves room for updating the evidence on other noise sources (air, railway traffic) and outcomes (quality of life, emotional and behavioral disorders) covered in the WHO review [4].

## 5. Conclusions

We found “very low” quality evidence that increasing exposure to road traffic noise may be associated with depression and anxiety. These findings were robust with no evidence for the available study-level moderators. Sensitivity analyses supported a linear relationship between noise and depression.

## Figures and Tables

**Table 1:** Descriptive characteristics of the studies included in the systematic review.

Publication	Country (Study)	Design	Analysis Sample	Mental Health Outcomes	Noise Exposure	Adjustments in Main Model
Floud et al. [20]	Greece, United Kingdom, Netherlands, Sweden, Italy, Germany (HYENA)	Cross-sectional (2004/6)	N = 4642; 45-70 years; 50.3% female; response rate 37-51%	Antidepressants (4.1%), anxiolytics (3.1%) (self-reported)	Modelled road traffic L_Aeq,24h_ at address; lived at the address for previous 5 years; 45-75 dB	Age, sex, education, BMI, alcohol, smoking, physical activity, country
Orban et al. [15]	Germany (HNR)	Prospective cohort (2000/3); ≈ 5.1 years follow-up	N = 3098; 45-75 years; ≈ 48% female; 55.8% response rate	Depression (antidepressants and/or self-reported scale) (9.2%)	Modelled road traffic L_den_ at each floor and façade; < 55 to > 70 dB	Age, sex, education, income, economic activity, area-level SES, traffic proximity
Seidler et al. [36]	Germany (NORAH)	Case-control (2010)	N = 77,295 cases (67.8% female) and 578226 controls (49.5% female); ≥ 40 years; 23% of the population	Depression – ICD-10: F32, 33, 34.1, 41.2 (register-based)	Modelled road traffic L_Aeq,16hr_; 40 to ≥ 70 dB	Age, sex, education/job title (where available), area-level SES, urban living
Okokon et al. [33]	Finland (HCREHS)	Cross-sectional (2015/16)	N = 5687/8; ≈ 55 ± 16 years; 57.4% female; 45-47% response rate	Anxiolytics (7%), antidepressants (7%) (self-reported)	Modelled road traffic L_den_ at most exposed façade; ≤ 45 to > 60 dB	Age, sex, income, marital status, employment, alcohol, smoking, physical activity, pet ownership
Zock et al. [65]	Netherlands	Cross-sectional (2013)	N = 4450; 40.5 years (0 to > 65); 50.9% female; 10% of all GP patients	Anxiety (4%), depression (4.5%) (register-based)	Modelled road traffic L_den_ at postcode-level; 61.2 dB (percentiles 58.3-64.0)	Age, sex, income, SES
Generaal et al. [31] ^1^	Netherlands (NESDA)	Cross-sectional (2004/7)	N = 2472/2560; ≈ 42 years (18-65); ≈ 66% female; 45% response rate	Anxiety, depression (diagnosed)	Modelled combined traffic L_den_; 55 (percentiles 53-57) ± 14.3 dB	Age, sex, education, income, municipality
Klompmaker et al. [30]	Netherlands (PHM)	Cross-sectional (2012)	N = 354,827; 19 to ≥ 65 years (43% ≥ 65); 54.6% female; 47% response rate	Anxiolytics (2%), antidepressants (7.3%), (register-based)	Modelled road traffic L_den_; 53.3 ± 7.5 dB	Age, sex, education, income, marital status, region of origin, occupation, alcohol, smoking, area-level SES, urbanization
He et al. [16]	Canada	Prospective pregnancy cohort (2000-2017); < 18 years follow-up	N = 140,456; < 25 to ≥ 35 years; 100% female; almost all of the population	Depression (0.7%)—ICD-9: 296.2, 296.3, 300.4, 309.28, 311; ICD-10: F32-34.1, 41.2 (diagnosed)	Modelled (LUR) combined traffic L_night_ at postcode-level; 62.4 ± 4.9 dB (49.2-84.9)	Age, pregnancy factors, comorbidity, area-level SES, neighbourhood walkability, time period, propensity score matching
Generaal et al. [32]	Netherlands (NEMESIS/ HELIUS/ NTR/ NESDA/ HOORN/ LASA/ NL-SH/ Generations)	Cross-sectional (2007-9/ 2011-15/ 2009-10/ 2004-7/ 2006-7/ 2005-6/ 2006-8/ 2009-15)	N = 6381/ 4634/ 11,388/ 2472/ 2667/ 1893/ 1575/ 1477. Age: 44 ± 13 (18-64)/ 46 ± 14 (18-70)/ 47 ± 13 (≥25)/ 42 ± 13 (18-65)/ 53 ± 17 (40-65)/ 71 ± 9 (55-85)/ 46 ± 12 (18-64)/ 35 ± 47 years. Female %: 55/ 54/ 62/ 66/ 53/ 55/ 64/ 100	Depression (diagnosed in NEMESIS/NESDA) and depressed mood (self-reported scale in the other studies); (6.4/ 7.3/ 6.3/ 5.2/ 5.1/ 5/ 5.8/ 4%)	Modelled combined traffic L_den_; 55±3.3/ 60 ± 2.5/ 54±5/ 55±3.2/ 54±2.3/ 53±3.3/ 54±3.6/ 56±5 dB	Age, sex, education, income
Leijssen et al. [34] ^2^	Netherlands (HELIUS)	Cross-sectional (2011/15)	N = 23,293; 44 years (18-70); 57.4% female; 55% response rate	Depressed mood (self-reported scale); (14.8%)	Modelled combined road traffic L_den_ at postcode-level; 45 to ≥ 70 dB	Age, sex, education, ethnicity, occupation, marital status, household composition, neuroticism, stressful life events, area-level SES, green/blue space, liveability

BMI: body mass index, L_Aeq,16hr_: daytime equivalent noise level, L_den_: day-evening-night noise level, L_dn_: day-night noise level, L_night_: night noise level, LUR: land use regression, SES: socioeconomic status. ^1^ Used only for the anxiety meta-analysis, ^2^ Used only for the categorical meta-analysis.

**Table 2:** Study-level characteristics as moderators of the effect of road traffic noise on depression under the quality effects model.

Study-Level Factor	N	OR/10 dB(A)	95% CI	I^2^ (%)	Moderation *p*-Value
Outcome assessment					
Diagnosis	5	1.04	0.92–1.17	28	Reference
Antidepressants	4	0.99	0.86–1.15	52	0.120
Self-report scale	6	1.03	0.73–1.46	47	0.676
Noise source					
Road traffic	6	1.04	0.97–1.10	70	Reference
Road traffic + other	9	1.07	0.90–1.28	45	0.386
Females % (continuous)	15	1.00	1.00–1.00	n/a	0.989
Mean/median age (continuous)	10	1.01	0.99–1.03	n/a	0.096
Minimum age (continuous)	14	1.00	1.00–1.00	n/a	0.150
Sample size (continuous)	15	1.00	1.00–1.00	n/a	0.548
Mean/median noise level (continuous)	11	1.00	0.98–1.02	n/a	0.838
Prevalence of depression (continuous)	14	0.98	0.93–1.03	n/a	0.386

N: number of estimates in the model, OR: odds ratio, CI: confidence interval, I^2^: heterogeneity statistic.

## Appendix A

**Table A1:** Bias criteria and scoring for studies included in the systematic review.

Bias Criteria
Publication type:
0 = Not peer reviewed;
1 = Peer reviewed article
Study design:
0 = Ecological;
1 = Cross-sectional;
2 = Case control;
3 = Cohort study
Selection of participants:
0 = No random sampling OR response rate less than 60% OR attrition rate higher than 20% OR no information provided;
3 = Participants randomly sampled from a known population AND response rate higher than 60%/most of source population sampled AND attrition rate less than 20% in follow-up studies
Sample representativeness:
0 = No information provided;
1 = Specific population group (e.g., narrow age range, disease status, socioeconomic status/education selection);
2 = Broader age range, no major selection;
3 = Reasonably representative of the general population, indicated by sampling method and/or provided comparison
Noise exposure quality:
0 = Objective method, low accuracy (e.g., postcode-level exposure) OR no information about resolution provided;
1 = Objective method, limited accuracy (land-use regression model, simple propagation modelling (engineering method) with poor traffic source data input, no validation measurements, no dwelling floor or noise barriers considered);
2 = Objective method, moderate accuracy (propagation modelling (engineering method) with validation measurements, considering noise barriers and/or dwelling floor);
3 = Objective method, high accuracy propagation modelling (scientific model), high quality traffic source data input, validation measurements with consideration of noise barriers and dwelling floor
Noise exposure timeframe:
0 = After study period OR no information provided;
1 = During study period;
2 = In addition: a previous assessment preceding the study period;
3 = 1 or 2 including a long-term residential history (duration of living)
Assessment of mental disorders:
0 = Self-report symptoms scale;
1= Self-reported diagnosis/ psychotropic medication use;
2 = Registry-based expert diagnosis/ psychotropic medication use;
3 = Clinical diagnosis/prescription
Confounding factors:
0 = None or only 1 important confounding factor considered (age or sex or education/socioeconomic status) OR no information provided;
1 = Confounding factors considered but at least 2 of the following are considered: age; sex; education/socioeconomic status;
2 = Consideration of all of the above confounders;
3 = Consideration of all of the above and area-level socioeconomic status/urbanicity;
4 = Consideration of all of the above and at least 1 of the following: ethnicity; marital status; both area-level socioeconomic status and urbanicity
Statistical analysis:
0 = No information provided;
1 = Flaws in or inappropriate statistical testing or interpretation of statistical tests that may have affected results (e.g., adjusting for mediators) OR transformation of effect estimates needed;
2 = Appropriate statistical testing and interpretation of tests; 3 = Specific advanced statistical model (multilevel analysis with appropriate data)
Additional bias:
0 = Other study or data extraction issues that may have led to bias;
3 = No other serious issues detected

**Table A2:** Study quality scores based on bias criteria.

Publication	Publication Type	Study Design	Selection of Participants	Sample Representativeness	Noise Exposure Quality	Noise Exposure Timeframe	Mental Disorders	Confounding Factors	Statistics	Bias	Overall Score
Floud et al. [20]	1	1	0	1	1	0	1	2	1	3	11
Orban et al. [15]	1	3	0	1	2	0	1	3	2	0^1^	13
Seidler et al. [36]	1	2	3	1	2	3	2	2	2	3	21
Okokon et al. [33]	1	1	0	3	2	2	1	2	1	0^1^	13
Zock et al. [65]	1	1	0	3	0	2	2	2	3	3	17
Generaal et al. [31]	1	1	0	1	2	1	3	2	3	0^1,2^	14
Klompmaker et al. [30]	1	1	0	1	1	2	2	4	2	0^1^	14
He et al. [16]	1	3	3	1	0	1	3	1	1	3	17
Generaal et al. [32]											
NEMESIS dataset	1	1	0	3	2	2	3	2	3	0^1,2^	17
HELIUS dataset	1	1	0	3	2	2	0	2	3	0^1,2^	14
NTR dataset	1	1	0	3	2	2	0	2	3	0^1,2^	14
NESDA dataset	1	1	0	1	2	2	3	2	3	0^1,2^	15
HOORN dataset	1	1	0	1	2	2	0	2	3	0^1,2^	12
LASA dataset	1	1	0	1	2	2	0	2	3	0^1,2^	12
NL-SH dataset	1	1	0	3	2	2	0	2	3	0^1,2^	14
Generations dataset	1	1	0	1	2	2	0	2	3	0^1,2^	12
Leijssen et al. [34] ^1^	1	1	0	3	0	2	0	4	1	0^2^	12

Score interpretation: higher scores indicate less bias. Bias domains scoring: See Table A1. ^1^ Transformation of effect estimate required. ^2^ Road traffic noise is not considered as an independent exposure.

**Table A3:** Data used for non-linear modelling of the exposure-response relationship between road traffic noise and depression.

Publication	OR	95% CI		L_den_ [dB(A)] ^1^
		Lower Bound	Upper Bound	
He et al. [16]	1	1	1	52.5
He et al. [16]	0.80	0.50	1.27	57.5
He et al. [16]	0.75	0.47	1.19	62.5
He et al. [16]	0.77	0.48	1.23	67.5
Leijssen et al. [34]	1	1	1	49.5
Leijssen et al. [34]	0.94	0.84	1.06	57
Leijssen et al. [34]	0.82	0.70	0.97	62
Leijssen et al. [34]	1.07	0.85	1.36	67
Leijssen et al. [34]	1.65	1.10	2.48	72
Okokon et al. [33]	1	1	1	42.5
Okokon et al. [33]	1.20	0.83	1.73	47.5
Okokon et al. [33]	1.13	0.78	1.64	52.5
Okokon et al. [33]	1.04	0.70	1.53	57.5
Okokon et al. [33]	1.32	0.91	1.90	62.5
Seidler et al. [36]	1	1	1	41.1
Seidler et al. [36]	1.02	1	1.06	46.1
Seidler et al. [36]	1.06	1.03	1.09	51.1
Seidler et al. [36]	1.09	1.06	1.12	56.1
Seidler et al. [36]	1.05	1.01	1.08	61.1
Seidler et al. [36]	1.12	1.08	1.16	66.1
Seidler et al. [36]	1.12	1.08	1.17	71.1
Seidler et al. [36]	1.17	1.10	1.25	76.1
Orban et al. [15]	1	1	1	52.5
Orban et al. [15]	1.19	0.86	1.65	57.5
Orban et al. [15]	1.52	1.11	2.07	62.5
Orban et al. [15]	1.19	0.85	1.68	67.5
Klompmaker et al. [30]	1	1	1	47.95
Klompmaker et al. [30]	0.99	0.95	1.04	50.65
Klompmaker et al. [30]	1.02	0.98	1.07	53.35
Klompmaker et al. [30]	0.98	0.93	1.02	56.7
Klompmaker et al. [30]	0.96	0.92	1.01	60.7

L_den_: day-evening-night noise level. ^1^ All exposure levels in studies using other noise metrics have been converted to L_den_.
